# Characterization of the ribonuclease activity on the skin surface

**DOI:** 10.1186/1479-0556-4-4

**Published:** 2006-05-29

**Authors:** Jochen Probst, Sonja Brechtel, Birgit Scheel, Ingmar Hoerr, Günther Jung, Hans-Georg Rammensee, Steve Pascolo

**Affiliations:** 1 Department of Immunology, Institute for Cell Biology, University of Tübingen, Auf der Morgenstelle 15, 72076 Tübingen, Germany; 2 Microbial Genetics, University of Tübingen, Auf der Morgenstelle 28, 72076 Tübingen, Germany; 3 Cure Vac GmbH, Paul Ehrlich Str.15, 72076 Tübingen, Germany; 4 Institute for Organic Chemistry, University of Tübingen, Auf der Morgenstelle 18, 72076 Tübingen, Germany

## Abstract

The rapid degradation of ribonucleic acids (RNA) by ubiquitous ribonucleases limits the efficacy of new therapies based on RNA molecules. Therefore, our aim was to characterize the natural ribonuclease activities on the skin and in blood plasma *i.e*. at sites where many drugs in development are applied. On the skin surfaces of *Homo sapiens *and *Mus musculus *we observed dominant pyrimidine-specific ribonuclease activity. This activity is not prevented by a cap structure at the 5'-end of messenger RNA (mRNA) and is not primarily of a 5'- or 3'-exonuclease type. Moreover, the ribonuclease activity on the skin or in blood plasma is not inhibited by chemical modifications introduced at the 2'OH group of cytidine or uridine residues. It is, however, inhibited by the ribonuclease inhibitor RNasin^® ^although not by the ribonuclease inhibitor SUPERase· In™. The application of our findings in the field of medical science may result in an improved efficiency of RNA-based therapies that are currently in development.

## Background

The presence of ribonucleases on human and rodent skin surfaces was described more than 40 years ago.[1,2] Subsequently their distribution within different skin layers was studied by different techniques.[3-5] However, the diversity, specificity and activity of extracellular (*i.e*. secreted or originating from dead cells) ribonucleases present on skin was never investigated.

However, information is available on extracellular ribonucleases expressed in internal human organs.[6] These enzymes belong to the RNaseA protein superfamily. Based on structural, catalytic and/or biological characteristics they can be classified into two major groups[7]: the pancreatic type (pt) and the non-pancreatic type (npt) ribonucleases. Human pt ribonucleases are similar to bovine pancreas RNaseA. They are active on poly(A) and double stranded RNA (dsRNA) and prefer as substrate poly(C) over poly(U). In contrast, npt ribonucleases are not active on poly(A) nor on dsRNA substrates and prefer poly(U) rather than poly(C) as substrate. At present, eight distinct human extracellular ribonucleases have been described at the genetic level. All of them are encoded by genes located on the long arm of chromosome14. At the protein level, five different ribonuclease activities have been described for human blood plasma. These ribonucleases range in size between 14 and 31 kDa.[8]

Extracellular ribonucleases are important in the formation of new blood vessels and thus tumor progression [9]. Indeed, Angiogenin that is the first identified tumor derived secreted angiogenic factor is an extracellular protein with a pt ribonucleolytic activity. This nuclease feature is necessary but not sufficient for angiogenin's angiogenic activity. However, the mechanisms of action of angiogenin and related poteins (angiogenins) on angiogenesis and in particular the role of the intrinsic RNAse activity, is still not clearly deciphered (for review see Strydom et al. [10]). For other extracellular ribonucleases it is suggested that they play a role in the prevention of infection by microbes [11,12] or RNA-viruses.[13] They might also control the hypothesized cell-to-cell communication mediated by the release and uptake of RNA by neighboring cells.[14] Finally, they may block unwanted activation of the immune system by dead cells which release RNA that, if not degraded, would stimulate antigen presenting cells (APC) through TLR-3, TLR-7 or TLR-8.[15-18]

The characterization of the extracellular ribonuclease activity has become again an attractive topic at the post-genomic era, where the development of safe gene therapies is needed for the transfer of basic research to the clinic. Plasmid DNA or recombinant viruses that were proposed as delivery vehicles for gene therapy approaches are associated to potential side effects and have uncontrolled half life.[19,20] As an alternative, mRNA, a nucleic acid with a controlled half life, is being evaluated in pre-clinical and clinical trials. Several mRNA-based immunization methods have been developed (reviewed in [21]): mRNA injected intradermally [22-26], mRNA entrapped in liposomes and injected subcutaneously or intravenously [27,28], mRNA loaded on gold particles and delivered intradermally by Gene-Gun [29] and mRNA transfected *in vitro *into APC.[30-33]

The quick degradation of mRNA by ubiquitous ribonucleases is one of the safety features of mRNA-based therapies. This process guaranties that the injected genetic information will be completely degraded and cleared from the body in a short time. The instability, however, puts an obvious limit on efficacy. Therefore, all mRNA-based therapies would benefit from the utilization of stabilized mRNA that have enhanced resistance towards ribonucleases contained in physiologic fluids, cell culture media and on the surface of the skin.

In order to gain more insights into the fundamental functions of extracellular ribonucleases, we investigated their diversity, their activity and their specificity. With the goal to enhance mRNA-based therapies, we also tested different strategies to stabilize the mRNA with regard to extracellular ribonuclease activity. We report here the characterization of the ribonuclease activity contained on the skin surface and in blood plasma and methods to inhibit them. Our results are relevant for applications in the field of mRNA-based therapies.

## Methods

### Animals

BALB/c mice were purchased from Charles River (Sulzfeld, Germany). The mice were not kept under special pathogen free conditions. All animal experiments were performed according to institutional and national guidelines.

### Preparation of ribonucleases

*Homo sapiens *skin surface ribonucleases were repeatedly isolated from one healthy individual by wetting an area of ~10 cm^2 ^pre-cleaned skin (sterilized and subsequently washed with soap and water) with 200–300 μl water for ~3 min. During this time the drop of water was several times pipetted up and down. For *Mus musculus*, skin surface ribonucleases were isolated by incubating an ear over night at 4°C in 200–300 μl water. Contact of water with the cut zone was avoided.

Protein content of skin surface preparations of both origins was below the detection limit for protein quantification by photometric measurements (Roti^®^-Nanoquant, Carl Roth, Karlsruhe, Germany). We observed only little variations in ribonuclease activity of different preparations as determined in degradation assays.

Peripheral blood from *Homo sapiens *and *Mus musculus *was collected in EDTA containing tubes to avoid coagulation. Blood plasma was separated by centrifugation for 6 min at 600 g and collected.

RibonucleaseA from *Bos taurus *pancreas was purchased from Roche (Mannheim, Germany) and dissolved in water to 10 mg/ml.

All preparations were aliquoted immediately and stored at -80°C.

### Ribonucleic acids

mRNA was produced by *in vitro *transcription with T7 RNA polymerase (T7-Opti mRNA kits, CureVac, Tübingen, Germany). Modified nucleotides were purchased from TriLink (San Diego, USA). All transcripts contained a poly(A) tail (70 bases long) and if not otherwise stated a 5'-cap structure. This cap structure was introduced during *in vitro *transcription: a fourfold excess of synthetic N7-Methyl-Guanosine-5'-Triphosphate-5'-Guanosine compared to GTP was used to guaranty that approximately 80% of the synthesized mRNA molecules started with a cap (whereas the remaining approximately 20% of the mRNA molecules started with GTP). Synthetic 18-mer RNA homopolymers were produced by CureVac using the phosphoramidite method. Poly(C) was purchased from Amersham (Freiburg, Germany)

### Zymogram

After denaturation at 95°C for 2 min in 1 × Laemmli loading buffer, samples were loaded on a SDS-PAGE where the 12, 5% stacking gel contained ~0, 6 mg/ml poly(C). Subsequently to electrophoresis (~2 h at 150 V), the gel was washed twice for 10 min with 25% (v/v) 2-propanol, 50 mM TrisHCl (pH7, 4) and 5 mM EDTA. The gel was scanned to document the position of the pre-stained molecular weight marker proteins (SeeBlue^® ^Plus2, Invitrogen, Karlsruhe, Germany). Then, it was further washed four times for 10 min with 50 mM TrisHCl (pH7, 4) and 5 mM EDTA (washing buffer). Thereafter, the gel was incubated at 37°C for 17 h in washing buffer supplemented with 150 mM NaCl. Ribonuclease activity was visualized by staining the gel with washing buffer supplemented with 0, 2%(w/v) toluidine blue O (Sigma, Munich, Germany) and destaining with washing buffer. For documentation the gel was scanned (GS-700, Biorad, Munich, Germany).

### Ribonuclease activity assay

Ribonuclease activity was assayed at 37°C in PBS (pH7, 2) by co-incubation of 0, 16 μg/ml of mRNA or 166 nM of 18-mer homopolymers and the indicated final dilution of ribonuclease preparations.

Reaction products were analyzed according to the following protocols: For mRNA, 6 μl samples were transferred to 6 μl formaldehyde loading buffer containing ethidium bromide (0, 01 mg/ml) and heat-denatured for 5 min at 80°C. The extend of mRNA digestion was analyzed by electrophoresis on formaldehyde agarose (FA) gels (1, 2%(w/v) agarose and 0, 65%(w/v) formalin in 1× FA buffer).

For RNA 18-mer homopolymers 6 μl samples were transferred to 6 μl formamide, heated for 5 min at 55°C, separated by urea-PAGE (42%(w/v) urea and 20%(w/v) acrylamide(29:1) in 1× TBE) and visualized by epiillumination of the gels on top of a thin layer chromatography plate.[34]

### Northern blot

The content of FA gels was blotted over night onto Hybond-N+ membranes (Amersham, Freiburg, Germany) by the capillary blot technique with 20× SSC as transfer buffer. After fixation (UV 1300 J/cm^2 ^plus backing 80°C for 2 h), membranes were equilibrated with hybridization buffer (5×SSC, 5×Denhardt's and 0, 5%(w/v) SDS) for 30 min at 50°C before the [γ-^32^P]-labeled 3'-probe (5'-GCA AGG AGG GGA GGA GGG-3', MWG-Biotech, Ebersberg, Germany) was added and incubation was continued over night. After repeated washing with decreasing salt (SSC) concentrations, the blot was exposed to a phosphor imager (PI) plate (Fujifilm, Düsseldorf, Germany). After documentation by scanning the PI plate (BAS-1500, Fujifilm) the blot was stripped by boiling in 0, 1% SDS (w/v) and then hybridized to the [γ-^32^P]-labeled 5'-probe (5'-TGA GCG TTT ATT CTG AGC TTC TGC-3', Thermo, Ulm, Germany). Some experiments were also carried out using first the 5'-probe and subsequently the 3'-probe. Densitograms were calculated with the Tina2.09d software (Raytest, Straubenhardt, Germany).

### Electroporation and FACS

Baby hamster kidney (BHK21) cells were grown to 80% confluence in cell culture medium (RPMI1640 supplemented with 100 U/μg/ml penicillin/streptomycin, 2 mM L-glutamine and 10%(v/v) FCS). Cells were harvested by trypsin-EDTA, washed once with cell culture medium and resuspended in PBS. Electroporation of 1–2 × 10^6 ^BHK cells in 4 mm cuvettes was performed at 250 V and 1050 μF in 200 μl PBS with 10 μg mRNA. After transfection, cells were immediately transferred to a cell culture vessel and allowed to grow for 15 h. They were harvested with trypsin-EDTA, fixed with 1%(w/v) formalin in PBS and analyzed by a FACSCalibur (BD, Heidelberg, Germany) flow cytometer and the CellQuest™ Pro software (BD).

### Ribonuclease inhibitors

The ribonuclease inhibitors RNasin^® ^and SUPERase· In™ were purchased from Promega (Mannheim, Germany) and Ambion (Huntingdon, UK). These inhibitors were added to ribonuclease activity assays before the addition of ribonucleases.

## Results

### Diversity of extracellular ribonucleases

**Figure 1:**
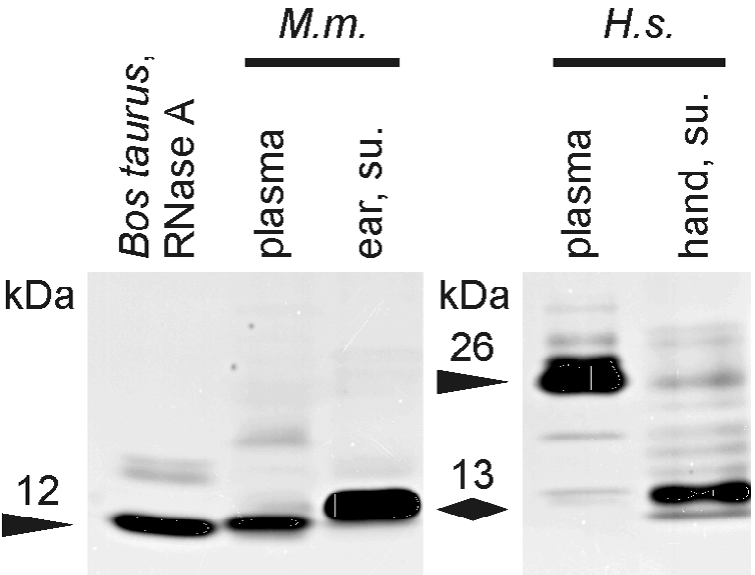
**RNA degrading proteins of skin surface preparations**. Zymogram (negative image). Ribonuclease activity in 10 μl preparation of blood plasma (prediluted 12, 5× in water) or ear surface (1×) from *Mus musculus *(*M.m*.) and of blood plasma (500×) or hand surface (1×) from *Homo sapiens *(*H.s*.) is shown and compared to the activity of 500 pg ribonuclease A from *Bos taurus *pancreas. Molecular weights of the most prominent bands were estimated by comparison to a molecular weight marker.

Human and mouse (BALB/c) extracellular skin ribonucleases (i.e. secreted or originating from dead cells) were obtained by applying nuclease-free water onto the skin surface. The recovered ribonuclease-contaminated solutions were aliquoted and stored at -80°C until use. The proteins contained in the preparations were separated by SDS-PAGE according to their size. An in-gel enzyme activity assay (zymogram) was subsequently performed. The activity profile of skin surface ribonucleases was compared to the one present in blood plasma. On such zymograms (Fig. 1) we found that the major ribonuclease activity on mouse skin is mediated by a protein of ~13 kDa in size. The protein responsible for the dominant ribonuclease activity in mouse blood plasma is slightly smaller (~12 kDa) than the one found on the skin. No or barely detectable additional ribonuclease activity was found for proteins of larger or smaller size. In contrast to the results obtained using mouse preparations, human preparations contained a broader spectrum of ribonuclease activities. The major ribonuclease activity on the human skin surface is very similar in size (~13 kDa) to the one on the mouse skin surface. Moreover, some sub-dominant ribonuclease activities can be observed for seven larger and one smaller protein. The dominant ribonuclease activity in human blood plasma is performed by a protein of ~26 kDa. Thus, in humans, the dominant ribonuclease activity is mediated by a different enzyme on the skin than in the blood plasma. Because most current therapies based on mRNA are delivered through the skin, we focused the rest of our study on the ribonuclease activity of skin surfaces.

### The ribonuclease activity on the skin surface is independent of the 5'-cap

**Figure 2:**
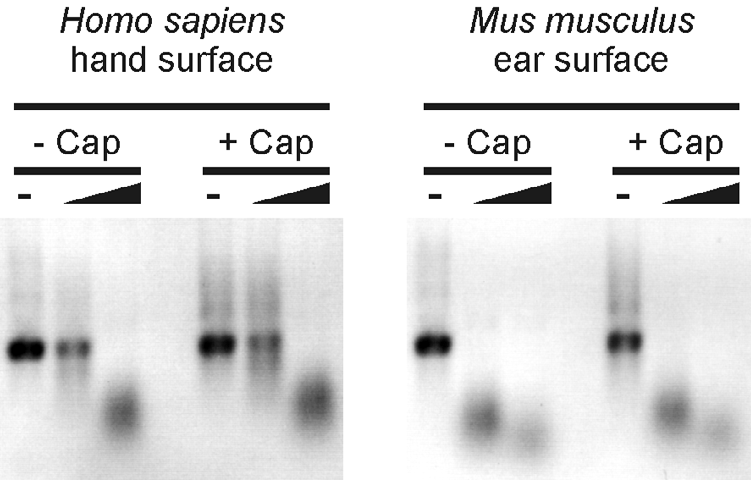
**Impact of a Cap structure at the 5'end of the substrate mRNA on the skin surface ribonuclease activity**. Ribonuclease activity assay (negative images). Non-capped (-Cap) or capped (+Cap) mRNA coding for enhanced green fluorescent protein (eGFP, ~1 kb) was incubated for 15 min at 37°C with increasing concentrations (indicated by the wedge) of preparations from *Homo sapiens *hand surface or *Mus musculus *ear surface: final dilution 20× or 4×, respectively. Samples without ribonucleases are indicated by a dash.

Besides its function in nuclear export and translation initiation the 5'-cap structure is important for the stability of intracellular mRNA in eukaryotic cells.[35] Therefore, we tested the capacity of the 5'-cap to protect the mRNA against extracellular ribonucleases. To this end, we produced capped and non-capped mRNA. Capped mRNA was made by adding a 4 fold excess of N7-Methyl-Guanosine-5'-Triphosphate-5'-Guanosine compared to GTP to the *in vitro *transcription reaction. Thus, approximately 80% of the transcribed mRNA molecules started with a cap. Purified transcripts were incubated with increasing concentrations of skin surface ribonucleases and the mRNA-degradation was analyzed by gel electrophoresis. The results shown in Fig. 2 indicate that capped and non-capped mRNA are degraded with the same kinetics. These experiments demonstrate that the 5'-cap does not influence the sensitivity of the mRNA to extracellular ribonucleases neither for mouse nor for human skin surface preparations. Thus, skin surface ribonucleases do not contain a dominant 5'-exonuclease activity or these enzymes can recognize equally well capped and non-capped mRNA.

### The ribonuclease activity on the skin surface is not dominantly of the exonuclease type

**Figure 3:**
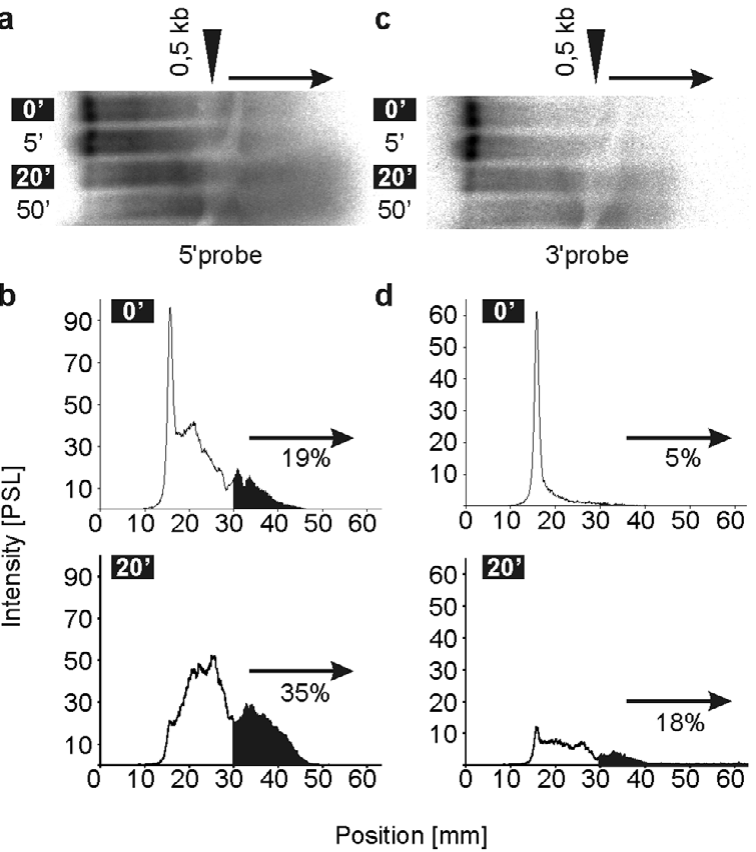
**Skin surface exoribonuclease activity**. Analysis of degradation fragments by Northern Blot. Capped β-galactosidase (lacZ, ~3, 5 kb) encoding mRNA was digested for the indicated time by ribonucleases from *Homo sapiens *hand surface (final dilution 4×) and detected by a probe binding close to the 5'-end (in A and B) or after stripping of the blot by a probe binding close to the 3'-end (in C and D) of the mRNA. The densitograms of bound radioactivity for the highlighted lanes (0' and 20') of the gels (A and C) are shown in the graphs (B and D). PSL means "photo-stimulated luminescence". Percent values given in B and D are the amount of radioactivity bound by fragments smaller than 0, 5 kb (filled area in B and D) compared to the activity bound by all fragments (filled and open area in B and D).

Ribonucleases are either endo- or exonucleases. Exonucleases have a predominant role for intracellular mRNA decay.[35,36] To determine whether the nuclease activity contained in extracellular ribonuclease preparations made from skin surface is of the 5'-exo, 3'-exo or endo-nuclease type, we performed ribonuclease activity assays followed by northern blots. Theoretically, if the mRNA is degraded from one end, no degradation fragments should hybridize with the probe specific for this end. Only the full length mRNA would be labeled. Experimentally, using either 5'- or 3'-specific oligonucleotide probes, we could detect a smear of degradation fragments (Fig. 3A and 3C). By quantification of the degradation fragments, we observed an increasing relative activity (percent values in Fig. 3B and 3D) of fragments smaller than 0, 5 kb (full length 3, 5 kb) and a lower total binding to the probes (course of the graphs in Fig. 3B and 3D) with increasing duration of digestion for both probes. Thus, the ribonucleases on the surface of human or mouse skin are not particularly degrading one end of the RNA. Instead, the activity is due to equally active 5'-and 3'-exonucleases and/or endonucleases.

### The ribonuclease activity at the skin surface is pyrimidine-specific

**Figure 4:**
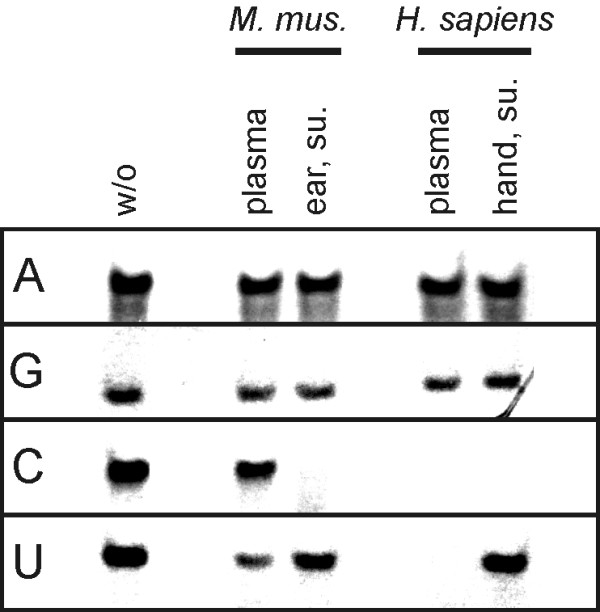
**Substrate specificity of skin surface ribonucleases**. Ribonuclease activity assay. 18-mer homopolymers of A, G, C or U were digested with ribonucleases of blood plasma (final dilution 400×) or ear surface (4×) from *Mus musculus *and with ribonucleases of blood plasma (400×) or hand surface (4×) from *Homo sapiens*.

To further characterize the ribonuclease activity in skin surface preparations we sought to determine its substrate specificity by using synthetic 18-mer homopolymers. Following incubation with skin surface preparations, the oligonucleotides were separated from their degradation products by urea-PAGE and visualized by the epiillumination technique. As shown in Fig. 4, poly(A) and poly(G) oligonucleotides are very resistant to the degradation by skin surface ribonucleases. On the contrary, poly(C) is readily degraded by mouse and human extracellular ribonucleases. Human blood plasma ribonucleases show a similar pattern of activity as human skin surface ribonucleases as far as degradation of poly(C) is concerned but are different as far as degradation of poly(U) is concerned: Skin surface ribonucleases do not degrade poly(U) although blood plasma ribonucleases do. This difference in substrate specificity between blood plasma and skin surface ribonucleases correlates with the zymogram results shown above (Fig. 1): the dominant skin surface ribonuclease activity is mediated by a different protein than the one mediating the dominant blood plasma ribonuclease activity. For mice, the ribonuclease activity in blood plasma is mainly specific for U while the skin surface ribonuclease activity is mainly specific for C. Here again this difference correlates with the size difference between the protein mediating the dominant ribonuclease activity in blood plasma and the one mediating the main ribonuclease activity on skin surface (Fig. 1). To conclude, both for mice and humans, the ribonuclease activity on the skin surface is specific for C. Instead (in mice) or additionally (in humans), some ribonuclease activity specific for U are contained in blood plasma.

### 2' modified mRNA have no increased resistance towards extracellular ribonucleases

**Figure 5:**
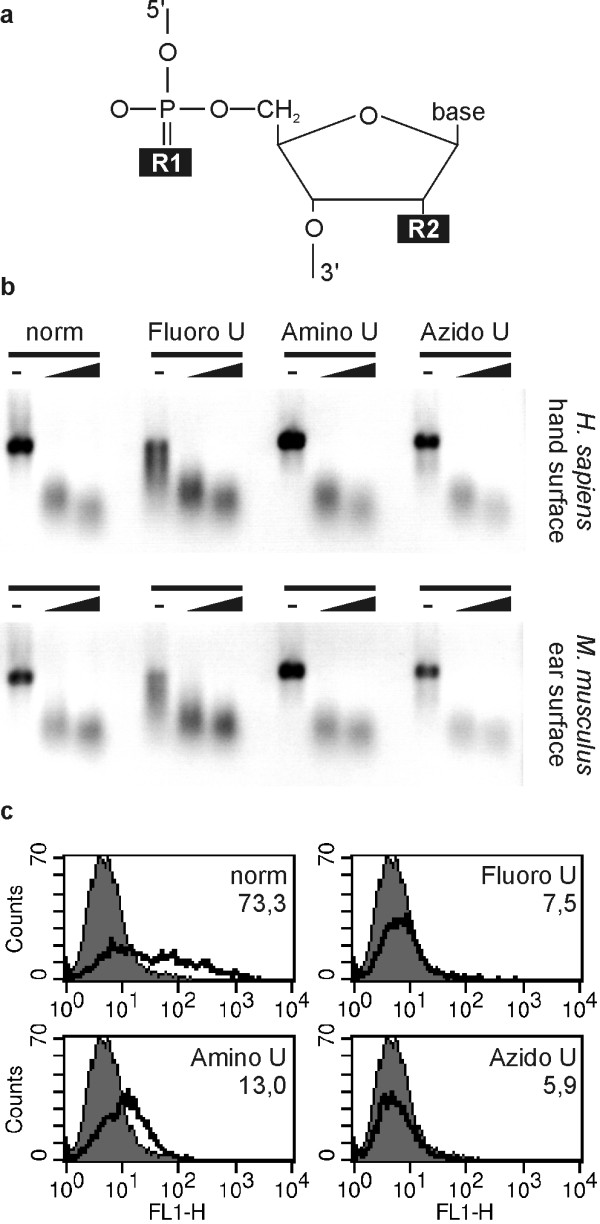
**Translation and stability (against skin surface ribonucleases) of cis-modified mRNA**. Ribonuclease activity assay and translation of mRNA. *In vitro *transcription of capped eGFP mRNA was performed with non-modified NTP (norm), or with 2'-fluoro-2'-deoxy UTP (Fluoro U), 2'-amino-2'-deoxy UTP (Amino U) or 2'-azido-2'-deoxyuridine (Azido U). See A) for the position (R2) of the substituted residue. B) Transcripts were incubated with ribonucleases from *Homo sapiens *hand surface (final dilution 4×) or *Mus musculus *ear surface (20×) at 37°C for increasing time (indicated by the wedge): 15 min or 60 min, respectively. Samples without ribonucleases are indicated by a dash and were incubated for 60 min at 37°C. Negative images. C) Transcripts (10 μg) were also used to electroporate BHK21 cells. Expression of eGFP was monitored in the FL1 channel by FACS analysis. For each transcript (open histograms) the expression is shown relative to non-transfected cells (filled histograms). Numbers indicate the mean of fluorescence intensity of the cells transfected with the different transcripts (for non-transfected cells the mean is 6, 1).

Since extracellular ribonucleases recognize pyrimidines (Fig. 4), an obvious strategy to improve the stability of the mRNA would be to produce mRNA that contains 2'-modified cytidines or uridines. To this end, the chemical modifications have to fulfill two criteria: (i) incorporation of modified nucleotides by the RNA polymerase during *in vitro *transcription and (ii) translation of the modified mRNA by ribosomes. In our hands, only 4 out of 10 tested nucleotides (cytidines or uridines with 2'-amino-2'-deoxy-, 2'-ara-, 2'-azido-2'-deoxy-, 2'-fluoro-2'-deoxy- or 2'-O-methyl sugar moieties, substitutions at position R2 in Fig. 5A) were successfully incorporated in mRNA molecules using either T7 or SP6 RNA polymerase: 2'-fluoro-2'deoxycytidine (data not shown), as well as 2'-fluoro-, 2'-amino-2' and 2'-azido-2'-deoxyuridine. Still, the quality of the *in vitro *transcription was affected: a large amount of abortive (short) mRNA could be seen on agarose gels, especially when transcribing long genes like LacZ (3.5 kb, data not shown). When using two modified nucleotides in the transcription reaction (2'-fluoro-2'deoxycytidine plus 2'-fluoro-2'deoxyuridine, for example) no full length mRNA product was obtained. Only one of the four modified mRNA was translated, albeit at low level compared to the natural non-modified mRNA, after transfection in BHK21 cells (Amino U, Fig. 5B). Moreover, none of these four different 2'-modified mRNA had an increased resistance towards skin surface ribonucleases (Fig. 5C). Thus, mRNA containing one nucleotide with a 2' modification are poorly generated by RNA polymerases, are poor templates for ribosomes *in vivo *and do not have increased resistance towards extracellular ribonucleases.

Alternatively to 2' modified nucleotides, sulfur substitutions at the phosphate group (R1, Fig. 5A) of pyrimidines might enhance mRNA stability. However, we obtained similar results as for 2' modified nucleotides (data not shown): poor transcription and no enhanced stability towards ribonucleases when using one or a combination of phosphorothioate nucleotide triphosphates.

### RNasin^® ^but not SUPERase· In™ protects mRNA from ribonuclease activity of skin surfaces

**Figure 6:**
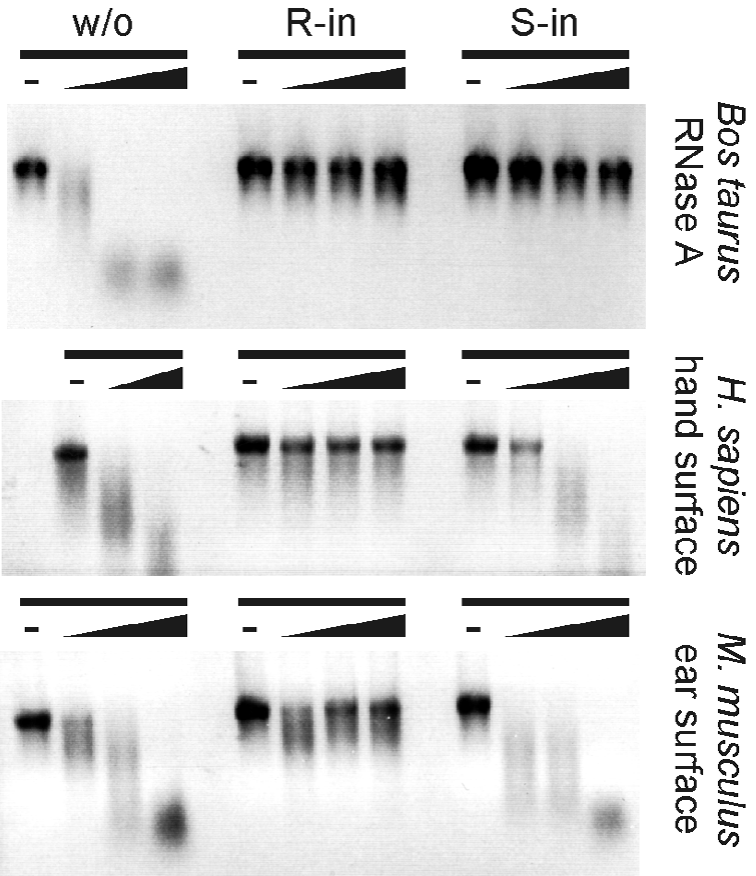
**Trans-protection of mRNA against skin surface ribonuclease activity**. Ribonuclease activity assay (negative images). Capped lacZ mRNA in the absence (w/o) or presence of the ribonuclease inhibitors RNasin^® ^(R-in, final concentration 1 U/μl) or SUPERase· In™ (S-in, 1 U/μl) was incubated with ribonucleases of *Homo sapiens *hand surface (final dilution 4×) or of *Mus musculus *ear surface (20×) or with 2, 5 pg/μl ribonuclease A from *Bos taurus *pancreas at 37°C for increasing time (indicated by the wedge): Samples were taken immediately after addition of ribonucleases as well as 15 min and 60 min after. Samples without ribonucleases are indicated by a dash and were not incubated at 37°C.

As an alternative to direct chemical modifications, mRNA can be protected from degradation by ribonuclease-inhibitors. One of the well known ribonuclease-inhibitors is diethyl pyrocarbonate (DEPC). DEPC is a highly reactive alkylating agent and therefore, very toxic. Consequently, it can not be used for the protection of mRNA in the context of mRNA-based therapies. Another class of widely used ribonuclease inhibitors consists of proteins. Among this class, RNasin^® ^is by far the best described. This 50 kDa protein was originally purified from human placenta. It binds with high affinity to ribonucleases of the RNaseA family forming a 1:1 complex [37]. Recently, a new protein capable of ribonuclease-inhibition was characterized: SUPERase· In™ (Ambion). SUPERase· In™ is reported to have a broader range of ribonuclease-inhibiting activity than RNasin^®^. We compared the two proteins for their ability to protect *in vitro *transcribed mRNA against ribonucleases contained in skin surface preparations. Therefore, the ribonuclease-inhibitors were mixed with the *in vitro *transcribed mRNA substrate before being incubated with the ribonucleases. Surprisingly, only RNasin^® ^could protect efficiently from ribonuclease activity (Fig. 6). SUPERase· In™ was as active as RNasin^® ^for the inhibition of purified RNaseA from *Bos taurus *pancreas but inefficient in preventing the degradation of mRNA by ribonucleases of the skin cell surface of *Homo sapiens *and *Mus musculus*. Thus, although SUPERase· In™ has a large spectrum of ribonuclease inhibition, it is not well adapted to block the natural extracellular ribonuclease activity of the skin. Besides, this experiment suggests that ribonucleases contained in the skin surface preparation are not dominantly of the pancreatic (RNaseA-like) type since in this case they should be equally inhibited by RNasin^® ^and SUPERase· In™.

## Discussion

Towards the characterization of the concerted extracellular ribonuclease activity (i.e. secreted or originating from dead cells), we first evaluated the number of proteins with different sizes capable of ribonuclease activity on the skin surface or in blood plasma (Fig. 1). Zymograms indicated that the skin surface contains one dominant ribonuclease activity mediated by a ~13 kDa protein. In humans, several sub-dominant ribonuclease activities are performed by 7 larger and one smaller protein. The ribonuclease activity of blood plasma is dominantly mediated by a protein of ~12 kDa in mice and ~26 kDa in humans. Further characterization of the ribonuclease activities on the skin surface indicated that they are not dominantly of a specific exonuclease type (5'-exo or 3'-exo, Fig. 3), are not impaired when the substrate contains a 5'-cap structure (Fig. 2), are specific for pyrimidines (Fig. 4) and can be efficiently inhibited by RNasin^® ^but not SUPERase· In™ (Fig. 6).

Moreover, using homopolymers as substrates, we found in all cases (mouse and human, skin surface and blood plasma) that the extracellular ribonucleases are specific for pyrimidines and that C is their preferred substrate (except for ribonucleases contained in mouse blood plasma where U is preferred, Fig. 4). This result has a great impact on the development of RNA-based drugs. Since a similar specificity was observed for the major ribonuclease extracted from mammalian's epidermis [38,39] we anticipate that the utilization of C-low RNA may be a method to increase the efficacy of RNA-therapies delivered transcutaneously, intradermally or subcutaneously.

We investigated whether the preference of extracellular ribonucleases for pyrimidines was exploited by viruses: a low U and C content in their transcriptome would be an advantage for their mRNA half life (especially when the genome is a RNA molecule). Comparing the mean (± standard deviation) C content of human mRNA (26, 5 ± 4, 3%) to the mean C content of RNA viruses (25, 1 ± 7, 3% for retro, 23, 1 ± 5, 4% for plus ssRNA and 19, 6 ± 2, 0% for minus ssRNA viruses) we cannot detect a clear tendency for a lower C content in RNA viruses. Thus, viruses do not appear to have evolved in order to resist extracellular ribonucleases.

In the context of mRNA-based therapies, a possible method to protect the nucleic acid against degradation by extracellular ribonucleases would be to modify pyrimidines, rendering them resistant to ribonucleases. Unfortunately, in our reaction conditions, most available UTP or CTP with a 2' modification were no substrates for *in vitro *polymerization with T7 or SP6 polymerase. 2'-fluoro substitutions were shown to be compatible with *in vitro *polymerization [40] but we failed to produce long mRNA containing modified U and modified C together. A single 2'-modified nucleotide (U or C) could not stabilize the mRNA sufficiently to resist extracellular ribonucleases while it abrogated translation *in vivo *(in transfected cells, Fig. 5). Thus, the available 2'-modified pyrimidines do not allow the generation of functional mRNA resistant to extracellular ribonucleases. Moreover (data not shown), neither the use of phosphorothioate modified cytidine [41] (sulfur for oxygen substitution at the phosphate residue, position R1 in Fig. 5A) nor the addition of poly(C) to the ribonuclease mixture (as a competitor for ribonuclease activity) did improve mRNA stability.

In contrast, the natural ribonuclease inhibitor RNasin^® ^was efficient in preventing the degradation of mRNA by extracellular ribonucleases (Fig. 6). RNasin^® ^was also more effective than SUPERase· In™ for the inhibition of the ribonucleases present on the skin surface. This result was unexpected since SUPERase· In™ has a larger reported spectrum of ribonuclease inhibition compared to RNasin^®^. Indeed, SUPERase· In™ may be more efficient than RNasin to inhibit ribonucleases in other applications. In the case of RNA protection against skin surface ribonucleases, RNasin^® ^might have some unknown relevant ribonuclease-specificity.

Our data suggest that mRNA used for therapies as an injected drug should be delivered together with RNasin^®^. RNasin^® ^being a human self protein, is not expected to have side effects: It should be catabolized naturally in a relatively short time, it should be not toxic for cells and, because it is a conserved self protein that is expressed in several organs [42], it should not trigger an immune response.

Although our studies document the activities of extracellular ribonucleases present on the skin, they do not provide an explanation for the role of such molecules. Some of the extracellular ribonucleases may originate from the cytosol of dead keratinocytes that constitute the skin surface. This seems to be unlikely since intracellular ribonucleases are mainly of the exonuclease type [35] and we could demonstrate that this is not the case for extracellular ribonucleases (Fig. 3). Besides, the characterization at the DNA level of genes coding for secreted (defined by the presence of a leader sequence) ribonucleases demonstrates that there must be a need in higher organisms for such activities at their surface. All three hypothesized roles of these ribonucleases on the skin (protection against foreign pathogens like RNA-viruses, prevention of the activation of the immune system by RNA released from dead cells or inhibition of cell-to-cell interactions through release-capture of RNA by neighboring cells) are not mutually exclusive. A role for RNA in cell-to-cell communication mediated by secretion and recapture of RNA by neighboring cells was originally suggested by Benner.[14] In line with this hypothesis we observed a lower content of ribonuclease activity in fast dividing tissues like tumors (data not shown and [43]).

Further studies are required to prove whether extracellular ribonucleases play indeed a role in the control of cell growth.

## Conclusion

RNases present at the skin surfaces recognize pyrimidines and are not inhibited by a 5'cap structure. As far as enzymatically produced messenger RNA are concerned, the replacement of natural nucleotides by chemically substituted ones is limited by the poor utilization of such analogs by RNA polymerases. Moreover, chemical modifications did not decrease RNase-sensitivity and they impaired translation. For protecting exogenous mRNA from RNases and keeping an efficient mRNA translation, we found that the best method is to mix non-modified, natural mRNA together with the protein RNAsin^®^. This is a simple method that can protect the extracellular therapeutic mRNA. Particularly in the context of mRNA-based vaccination, such a trans-protection of the mRNA thanks to additional RNAsin^® ^can be foreseen as safe method to improve the mRNA'as half life, thus its penetration in cells and thereby the efficacy of the vaccine.

## Authors' contributions

JP performed most of the assays and drafted the manuscript

SB performed the experiments presented in figure 2 and 3

BS participated in the set-up of the experiments

IH, GJ and HGR contributed to the intellectual development of this research and to its financial support

SP conceived and supervised this project.
